# Factors associated with willingness to use oral pre-exposure prophylaxis (PrEP) in a fisher-folk community in peri-urban Kampala, Uganda

**DOI:** 10.1186/s12889-022-12859-w

**Published:** 2022-03-09

**Authors:** Bashir Ssuna, Anne Katahoire, Mari Armstrong-Hough, Dennis Kalibbala, Joan N. Kalyango, Flavia Matovu Kiweewa

**Affiliations:** 1 Department of Epidemiology and Biostatistics, Makerere College of Health Sciences, P.O. Box 21696, Kampala, Uganda; 2 Uganda Tuberculosis Implementation Research Collaboration (U-TIRC), P.O. Box 21696, Kampala, Uganda; 3 Makerere College of Health Sciences, Child Health and Development Centre, Kampala, Uganda; 4 Department of Social and Behavioral Sciences, Department of Epidemiology, New York University School of Global Public Health, New York, USA; 5 Makerere University-John Hopkins University Research Collaboration (MU-JHU), Kampala, Uganda

**Keywords:** Pre-exposure prophylaxis, Fisherfolk, Acceptability, Key populations

## Abstract

**Background:**

The World Health Organization (WHO) recommends the use of pre-exposure prophylaxis (PrEP) in key populations at elevated risk for exposure to HIV. If used effectively, PrEP can reduce annual HIV incidence to below 0.05%. However, PrEP is not acceptable among all communities that might benefit from it. There is, therefore, a need to understand perceptions of PrEP and factors associated with willingness to use PrEP among key populations at risk of HIV, such as members of communities with exceptionally high HIV prevalence.

**Objective:**

To examine the perceptions and factors associated with willingness to use oral PrEP among members of fishing communities in Uganda, a key population at risk of HIV.

**Methods:**

We conducted an explanatory sequential mixed-methods study at Ggaba fishing community from February to June 2019. Survey data were collected from a systematic random sample of 283 community members in which PrEP had not been rolled out yet by the time of we conducted the study. We carried out bivariate tests of association of willingness to use PrEP with demographic characteristics, HIV risk perception, HIV testing history. We estimated prevalence ratios for willingness to use PrEP. We used backward elimination to build a multivariable modified Poisson regression model to describe factors associated with willingness to use PrEP. We purposively selected 16 participants for focus group discussions to contextualize survey findings, analysing data inductively and identifying emergent themes related to perceptions of PrEP.

**Key results:**

We enrolled 283 participants with a mean age of 31 ± 8 years. Most (80.9%) were male. The majority of participants had tested for HIV in their lifetime, but 64% had not tested in the past 6 months. Self-reported HIV prevalence was 6.4%. Most (80.6, 95%CI 75.5–85.0) were willing in principle to use PrEP. Willingness to use PrEP was associated with perceiving oneself to be at high risk of HIV (aPR 1.99, 95%CI 1.31–3.02, *P* = 0.001), having tested for HIV in the past 6-months (aPR 1.13, 95%CI 1.03–1.24, *P* = 0.007), and completion of tertiary education (aPR 1.97, 95%CI 1.39–2.81, *P* < 0.001). In focus group discussions, participants described pill burden, side-effects and drug safety as potential barriers to PrEP use.

**Conclusions and recommendations:**

Oral PrEP was widely acceptable among members of fishing communities in peri-urban Kampala. Programs for scaling-up PrEP for fisherfolk should merge HIV testing services with sensitization about PrEP and also increase means of awareness of PrEP as an HIV preventive strategy .

## Introduction

Of the 1.7 million new HIV infections that occurred globally in 2019, 62% were among key populations and 28% of these infections occurred in key populations of East and Southern Africa [1]. In Uganda, as in many settings, the HIV burden is mostly concentrated in key populations [2]. In 2019, the average national HIV prevalence rate among adults aged 15–49 years was 5.8% [3], while 15–40% of adult fisher folk were living with HIV [4]. Fisherfolk in Uganda are at elevated risk of exposure to HIV due to high degrees of mobility, poor access to information about HIV, and limited access to HIV prevention resources, including oral pre-exposure prophylaxis (PrEP) [5].

Daily use of oral PrEP is reported to reduce the risk of HIV transmission through sex by 75 to 99% [6, 7]. Based on this evidence and following the 2015 WHO recommendation [8], the Ministry of Health rolled out oral PrEP (300 mg of Tenofovir daily or 300 mg of Lamivudine daily) to key populations in Uganda, including fisherfolk beginning in August 2017 [9]. To date, an estimated 21,000–22,000 members of key populations in Uganda including but not limited to sex workers, long-distance truck drivers, barmaids, and discordant couples have accessed oral PrEP [10]. However, studies show that poor user knowledge, negative community perceptions toward the drug, stigma, cultural beliefs, and low perceived risk of HIV transmission continue to dampen acceptability of oral PrEP leading to poor adherence and reduced effectiveness [11–13]. Studies outside Uganda in low and high-income countries have found levels of awareness of PrEP as low as 29.7% among HIV key populations, and acceptability ranging from 35.4 to 64.4% [14, 15].

Though fishing communities are among the key populations targeted for oral PrEP, to date, the majority of these communities have lacked comprehensive knowledge on HIV prevention, have misconceptions about HIV transmission and about 35% use the common HIV preventive strategies like condoms [16, 17]. We aimed study to explore the dynamics of PrEP acceptability among fisherfolk and to identify the factors necessary to improve PrEP implementation process in order to inform countrywide PrEP rollout in Uganda.

## Materials and methods

We carried out a cross-sectional study using an explanatory sequential mixed-methods design, integrating survey data and focus group discussions collected from 5th February to 4th June 2019.

The study was conducted in Ggaba, a semi-urban landing site on Lake Victoria located in Makindye division of Kampala district, the capital of Uganda. Ggaba has a population of around 17,000 people [18] with the major economic activity being fishing. The population is highly transient and has a high HIV transmission rate of 3.39 per 100 person-years at-risk compared to 0.46 per 100 person-yers in the general population [19–21].

We included adults aged 18 and above who had been residents at the Ggaba landing site for at least 3 months and provided informed consent to participate in the study. We excluded individuals who were too ill to participate in the study.

For the primary quantitative research question, which aimed to determine the proportion of individuals willing to use oral PrEP. For this component, we used one proportion (Kish Leslie formula of 1965) alpha was set at 0.05, Z_α_ at 1.96, the estimated proportion (d) as 88.8% and design effect at 2. For associated factors, we used a comparison of two proportions (Fleiss formula) [22]. We set power at 80% (Z_β_ = 0.84), alpha at 0.05 (Z_α_ = 1.96) and based on the previous studies by Jayakumaran et al., 2016 and Frankis et al., 2016 [23], we used the gender factor to estimate a minimum sample size of 283 participants.

We used a systematic sampling method for the survey. We estimated a population of 1000 adults at the landing site and generated a sampling fraction of four with a random number between one to four used to generate the sampling interval of three. We, therefore, included every fourth adult was included in the survey beginning with the third adult until the sample size was attained.

Trained interviewers administered a semi-structured questionnaire with both open and closed-ended questions using Open Data Toolkit (ODK) in English and/or Luganda, the local language. The questionnaire was adopted from previous literature about acceptability of PrEP use and pretested on 15 random participants who were not included in the study. Acceptability was defined as self-reported willingness to use PrEP when provided, and was measured using “Yes” and “No”.

The qualitative component of the study consisted of focus group discussions (FGD) using maximum variation purposive sampling to explore acceptability and perceptions of oral PrEP. To facilitate exploration of disparate views and generate point-counterpoint discussion and resolutions, we used FGDs for general discussion on the subject. We ensured relative homogeneity in age, sex, and duration of stay at the landing site when recruiting the 16 participants for each of the two FGDs (8 per FGD). FGD data were collected using a focus group moderator guide, conducted in the local language, audiotaped and later transcribed and translated to English by the PI (BS) as part of standard operating procedures such that they have retained their meaning.

### Statistical analysis

We produced descriptive statistics such as proportions and mean ± standard deviations for all variables. The primary outcome was willingness to use oral PrEP. Willingness to use PrEP was measured as a proportion with its 95% confidence interval. A modified Poisson regression was used to estimate the prevalence ratios (PR) for bivariate and and adjusted prevalence ratios (aPR) for multivariate analysis. We used backward elimination method after assessing for assumptions of multicollinearity (by estimating VIF and leverage) and outliers. The adjusted prevalence ratios were assessed for confounding by 10% change in the crude and adjusted prevalence ratios. Analyses were done using Stata Version 15.1/MP.

The qualitative component of the study consisted of focus group discussions (FGD) using maximum variation purposive sampling to explore acceptability and perceptions of oral PrEP using Atlas.ti 8.3 software.. The lead author (BS) read all transcripts and used an inductive, iterative approach to analyze their content, applying three cycles of coding according to the study purpose statement. Emerging themes on perceptions about PrEP were grouped and discussed with other authors (MAH, AK) between each coding cycle.

## Results

### Description of study participants

Of the 283 participants, 80.9% were male and 82.7% were employed (Table 1). The mean age of participants was 31 ± 8 years. Self-reported HIV prevalence was 6.4 and 64% had not tested in the past 6 months, while 62.4% had concerns about acquiring HIV.

**Table 1:** Sociodemographic characteristics of the 283 participants from a fisherfolk community in Kampala

Characteristic	Frequency (n)	Proportion (%)
**Age**		
18–39 (Young adult)	232	82.0
40–62 (Middle aged)	51	18.0
**Gender**		
Male	229	80.9
Female	54	18.1
**Location**		
Semi-urban	271	95.8
Rural	12	4.2
**Education**		
None	39	13.8
Primary	89	31.5
Secondary	145	51.2
Tertiary	10	3.5
**Employment status**		
Employed	234	82.7
**Marital status**		
Single	111	39.2
Married	145	51.2
Casual	1	0.4
Divorced	21	7.4
Widowed	5	1.8
**HIV status awareness**		
Yes	207	73.1
**HIV test in the past 6 months**		
Yes	102	36.0
**Self-reported HIV status**		
Negative	188	66.4
Positive	18	6.4
Not-aware	77	27.2
**Condom use**		
Always	50	17.7
Sometimes	125	44.2
Never	108	38.1
**HIV risk perception**		
No	36	12.7
Yes	247	87.3
**HIV infection concern**		
Not really	36	12.7
Yes	182	64.3
Sometimes	65	23.0

### Factors associated with willingness to use PrEP

A high proportion (80.6, 95% CI 75.5–85.0) of participants reported that they were willing to use PrEP if it were provided.

In bivariate analyses we used willingness to use PrEP as the outcome, persons with age between 40 and 62 years was associated with 36% less willing to use PrEP compared to those between 18 and 39 years (crude PR = 0.64, *P* = 0.001), participants in semi-urban location were less willing to use PrEP compared to those from urban (crude PR =0.51, *P* = 0.047), and primary education level was associated with twice more willingness to use PrEP compared to no education (crude PR =2.03, *P* < 0.001), secondary education was associated with 2.15 times more willingness to use PrEP compared to no education (crude PR = 2.15, *P* < 0.001) while tertiary education was associated with 2.44 times more willingness to use PrEP compared to no education (crude PR = 2.44, *P* < 0.001) -(Table 2). Those who reported regularly testing for HIV were more likely to say they were willing to use PrEP compared to who tested irregularly (92.2% vs 74.0%, *P* < 0.001). The proportion of men who were willing to use PrEP was higher than that of women (83.0% vs 70.4%, *P* = 0.078). Having tested for HIV in the last 6 months was associated with 24% more willingness to use PrEP compared to those who did not test (crude PR = 1.24, *P* < 0.001), Positive HIV status was associated with 88% less willingness to recommend PrEP use compared to HIV negative individuals (crude PR = 0.12, *P* = 0.002), un-aware of HIV status was associated with 20% less willingness to recommend PrEP use compared to HIV negative individuals (crude PR = 0.12, *P* = 0.002). Participants having perception of HIV risk were 2.41 times more willing to use PrEP compared to those without (crude PR = 2.41, *P* < 0.001), participants who always had concern about HIV had 3.71 times more likelihood to use PrEP compared to those who did not (crude PR = 3.71, *P* < 0.001), Never using condom was associated with 19% less likelihood to use PrEP compared to those who always used (crude PR = 0.81, *P* = 0.002) while who sometimes used condoms had 11% less ikelihood to use PrEP compared to those who always used, and awareness of PrEP was associated with 15% more willingness to use PrEP compared to those who were not aware of PrEP (crude PR = 1.15, *P* = 0.024).

**Table 2:** Bivariate analysis for sociodemographic factors associated with willingness of PrEP in a fishing community

Characteristic	Total	Willingness to use PrEP		Crude PR	***P***-value
		Unwilling	Willing	[95% CI]	
**Age**					
18–39 (Young adult)	232	200 (86.2)	32 (13.8)	1.00	
40–62 (Middle aged)	51	28 (54.9)	23 (45.1)	0.64 [0.49–0.82]	0.001**
**Location**					
Semi-urban	271	48 (17.7)	223 (82.3)	1.00	
Rural	12	7 (58.3)	5 (41.7)	0.51 [0.26–0.99]	0.047*
**Education**					
None	39	23 (59.0)	16 (41.0)	1.00	
Primary	89	15 (16.9)	74 (83.2)	2.03 [1.37–2.99]	< 0.001**
Secondary	145	17 (11.7)	128 (88.3)	2.15 [1.47–3.15]	< 0.001**
Tertiary	10	0	10 (100)	2.44 [1.67–3.55]	< 0.001**
**Employment status**					
Not employed	49	8 (16.3)	41 (83.7)	1.00	
Employed	234	47 (20.1)	187 (79.9)	0.96 [0.83–1.10]	0.519
**Marital status**					
Not married	137	27 (19.7)	110 (80.3)	1.00	
Married/in a casual relationship	146	28 (19.2)	118 (80.8)	1.01 [0.90–1.13]	0.911
**Gender**					
Male	229	39 (17.0)	190 (83.0)	1.00	
Female	54	16 (29.6)	38 (70.4)	0.85 [0.71–1.02]	0.078
**HIV status awareness**					
No	76	16 (21.1)	60 (79.0)	1.00	
Yes	207	39 (18.8)	168 (81.2)	1.03 [0.90–1.17]	0.685
**HIV test in the past 6 months**					
Yes	102	8 (7.8)	94 (92.2)	1.24 [1.12–1.38]	< 0.001**
**Self-reported HIV status**					
Negative	188	18 (9.6)	170 (90.4)	1.00	
Positive	18	16 (88.9)	2 (11.1)	0.12 [0.03–0.46]	0.002*
Not-aware	77	21 (27.3)	56 (72.7)	0.80 [0.70–0.93]	0.003*
**Condom use**					
Always	50	4 (8.0)	46 (92.0)	1.00	
Sometimes	125	23 (18.4)	102 (81.6)	0.89 [0.79–1.01]	0.044*
Never	80	28 (25.9)	80 (74.1)	0.81 [0.70–0.92]	0.002*
**HIV risk perception**					
Yes	247	32 (13.0)	215 (87.0)	2.41 [1.56–3.74]	< 0.001**
**HIV infection concern**					
Not really	36	27 (75.0)	9 (25.0)	1.00	
Yes	182	13 (7.1)	169 (92.9)	3.71 [2.10–6.56]	< 0.001**
Sometimes	65	15 (23.1)	50 (76.9)	3.08 [1.72–5.51]	< 0.001**
**PrEP awareness**					
Yes	153	22 (14.4)	131 (85.6)	1.15 [1.02–1.29]	0.024*

HIV status, HIV infection concern, and condom use were dropped at multivariate due to multicollinearity

In the final multivariable model, three factors were significantly associated with willingness to use PrEP among fishing communities: higher levels of education, perceived risk for HIV, and history of testing for HIV within the last 6 months (Table 3). Compared to those with no formal education, participants who had attained tertiary education were nearly twice as likely to say they were willing to use PrEP (aPR 1.97, 95%CI 1.39–2.81, *p* < 0.001) while participants who had attained secondary education were 1.72 times as willing to use PrEP (aPR 1.72, 95%CI 1.22–2.44, *p* = 0.002) and those with primary education were 1.61 times more willing to use PrEP (aPR 1.61, 95%CI 1.12–2.29, *p* = 0.009). Compared to those who had not tested for HIV in the past 6 months, participants who had tested were 13% more willing to use PrEP (aPR 1.13, 95%CI 1.03–1.24, *p* = 0.007). Participants who perceived themselves at a risk of getting HIV were almost twice more willing to use PrEP than those who had no HIV risk perception (aPR 1.99, 95%CI 1.31–3.02, *p* = 0.001).

**Table 3:** Multivariate analysis for factors associated with willingness of PrEP in a fishing community

Characteristic	Crude PR	Adjusted PR	***P***-value
	[95% CI]	[95% CI]	
**Age**			
18–39 (Young adult)	1.00	1.00	
40–62 (Middle aged)	0.64 [0.49–0.82]	0.81 [0.65–1.01]	0.054
**HIV test in the past 6 months**			
No	1.00	1.00	
Yes	1.24 [1.12–1.38]	**1.13 [1.03–1.24]**	0.007*
**Location**			
Semi-urban	1.00	1.00	
Rural	0.51 [0.26–0.99]	0.71 [0.35–1.44]	0.341
**Education**			
None	1.00	1.00	
Primary	2.03 [1.37–2.99]	**1.61 [1.12–2.29]**	0.009*
Secondary	2.15 [1.47–3.15]	**1.72 [1.22–2.44]**	0.002*
Tertiary	2.44 [1.67–3.55]	**1.97 [1.39–2.81]**	< 0.001**
**HIV risk perception**			
No	1.00	1.00	
Yes	2.41 [1.56–3.74]	**1.99 [1.31–3.02]**	0.001*
**PrEP awareness**			
No	1.00	1.00	
Yes	1.15 [1.02–1.29]	1.05 [0.94–1.16]	0.405
**Gender**			
Male	1.00	1.00	
Female	0.85 [0.71–1.02]	0.86 [0.73–1.01]	0.061

Statistically significant result *P-value < 0.05, **P-value< 0.001

Perceptions of PrEP: Focus Group ResultsIn focus group discussions which included 8 men and 8 women between 19 and 40 years of age, 50% of which had at least secondary education, fisherfolk explained the reasons behind the high acceptability of PrEP. Participants favored the introduction of PrEP because they believed it would protect them against HIV. Some participants described it as a good drug that they anticipated could be used as a means of saving their lives. Two ladies from the landing site explained,

“The drug should be provided to private hospitals because we are badly off on these islands. But if you bring the drug you would have helped us a lot.” **(R4/FGD3).**

“…. if it’s saving my life, I have to take it so that I defend my life.” **(R2/FGD3).**

Another male fisherman explained;

“I was happy when I heard about the drug because it will help us most especially adolescents who are sexually active.” **(R8/FGD2).**

However, participants expressed concerns about adverse effects from the drug, which they perceived to be likely. They anticipated that such side effects would stop them from carrying out their normal daily activities. On lady explained that such side effects could decrease motivation to continue using PrEP:

“Maybe if we use it makes us dizzy or when it exhausts your energy, you may say that am not sick why I am bothering to take it, but if it has no problem it brings to your body then I just swallow it and I eat my food.” **(R5/FGD3).**

Others were concerned about the long-term effects of a preventive medication. While asking them about the major concerns about the drug, another explained;

“… doctor, this is the reason why we may not take it, they told us that when you swallow that drug when you don’t have the disease you get cancer inside the stomach especially where that drug sits because it doesn’t have what to treat in your body.” **(R4/FGD3).**

Fishermen were also concerned about possible changes in lifestyle PrEP might introduce. They explained;

“I was told it makes you lose your appetite, loose sleep when you are start taking it.” **(R8/FGD3).**

“If they are to stop us from other foods and drinks like alcohol.” **(R4/FGD3).**

## Discussion

In this expanatory-sequential study, most fisherfolk said they were willing to use oral PrEP. This is consistent with previous studies assessing the acceptability of PrEP among other key populations in East and Southern Africa [24]. In a discrete choice experiment to assess the acceptability and potential uptake of PrEP, others have also found oral PrEP to be highly acceptable in principle among fishing communities in Uganda [25].

In our study, PrEP was most likely to be acceptable among those who perceived themselves as being at high risk of exposure to HIV. This link between risk perception and acceptability to use PrEP also emerged in FGDs, where participants described their communities as being “badly off” with regard to HIV. High self-reported HIV risk perception among key population communities [26] could explain the high willingness to use oral PrEP among key populations.

We further found that having tested HIV in the past 6 months was associated with a 13% increase in likelihood of being willing to use PrEP. Similarly, perceiving oneself to be at risk of HIV infection was associated with a doubling of the likelihood of being willing to use PrEP compared to those who did not perceive themselves to be at risk. These findings are consistent with studies also done in other key populations like MSM in Mexico, Peru and Brazil [27]. In focus group discussions, participants perceived PrEP to be protective and necessary to defend their lives from HIV. This suggests that people in fishing communities who perceive themselves as being at risk of getting HIV and have regular HIV checkups are more likely to accept PrEP if provided to them. Indeed, we found regular HIV testing to be a predictor of PrEP acceptance in survey responses.

We also found that respondents who had completed primary education were 61% more likely to express willingness to use PrEP compared to those with no formal education. There was a stepwise relationship between educational attainment and willingness to use PrEP: secondary-level was associated with 72% increase in willingness while the tertiary level was associated with 97% increase in willingness. This is likely because those who were more educated had greater access to information and a better understanding of HIV risks, rendering them less prone to misconceptions from peers [28, 29]. Better understanding of oral PrEP may increase willingness to use it. Therefore, interventions to educate the communities on PrEP may be of importance to facilitate acceptance of the drug. This finding is consistent with studies conducted in other key populations where those who were more educated believed that PrEP would reduce HIV risk to their partners and would accept it and recommend it [12, 15, 30].

From our FGDs we found that respondents had concerns about the effects of PrEP to their bodies and how it could affect their daily activities. Such perceptions included drug side effects, negative perceptions about PrEP being carcinogenic and lifestyle modifications like stoping alcohol. These negative perceptions could be as a result of lack of access to PrEP educational materials with correct information and lack of PrEP promotion campaigns in the area which could have benefited the population. These findings are consistent with studies documenting perceptios about PrEP in key population [29, 31].

Our study has some limitations. We measured only hypothetical acceptability of oral PrEP rather than preferences after the use of the actual tablets. We had a mismatch of male to female ratio, more males participants were recruited. Self-reported HIV status: We may have included HIV positive individuals since some had not tested in the last 6 months and could have gotten infection in that time.

Our study also has several strengths. First, the study participants were systematically sampled from its fishing sites to ensure high representativeness of the community. Second, we sequentially employed both quantitative and qualitative methods to gain a more thorough understanding of the factors associated with willingness to use oral PrEP among this community.

## Conclusions and recommendations

Oral PrEP was widely acceptable among members of fishing communities in peri-urban Kampala. From the FGDs, we also learned that PrEP is considered as a life saver to the fishing communities due to their lifestyle. Programs for scaling-up PrEP for fisherfolk should merge HIV testing services with sensitization about PrEP,address awareness of PrEP and misconceptions.

This work was presented in part at the 15th Joint Annual Scientific Health Conference (JASH), Hotel Africana, Uganda, Abstract-UAN-025.

## Data Availability

The datasets used and analyzed during this study are available from the corresponding author on reasonable request because it involved other key population groups which are criminalized in Uganda.

## Abbreviations

HIV: Humman Immunodeficiency Virus
PrEP: Pre-exposure Prophylaxis
SOMREC: School of Medicine Research and Ethics Committee
WHO: World Health Organization

## References

[1] UNAIDS (2020). Global HIV & AIDS statistics — 2020 fact sheet.

[2] UAC (2018). ACCELERATION OF HIV PREVENTION IN UGANDA: A road map towards zero new infections by 2030.

[3] UNAIDS. Uganda [Internet]. 2020 [cited 2021 Feb 12]. Available from: https://www.unaids.org/en/regionscountries/countries/uganda

[4] Uganda AIDS Commission, MoH Uganda. The Uganda HIV and AIDS Country Progress Report [Internet]. 2016. Available from: uac.go.ug/sites/default/files/JAR%202016.pdf.

[5] International organization for migration (2014). Ugandan fishing communities at high risk of HIV / AIDS: IOM - Uganda [Internet]. ReliefWeb.

[6] CDC (2019). Pre-Exposure Prophylaxis (PrEP) | HIV Risk and Prevention | HIV/AIDS | CDC.

[7] Baeten JM, Donnell D, Ndase P, Mugo NR, Campbell JD, Wangisi J (2012). Antiretroviral prophylaxis for HIV prevention in heterosexual men and women. N Engl J Med.

[8] WHO. WHO | Pre-exposure prophylaxis: WHO; 2015. [cited 2019 Nov 7]. Available from: http://www.who.int/hiv/topics/prep/en/

[9] Uganda MH (2018). Consolidated guidelines for the prevention and treatment of HIV and aids in UGANDA.

[10] PrEPWatch. Uganda: A snapshot of PrEP scale-up, registration and resources for Uganda. [Internet] PrEPWatch 2020 [cited 2020 Apr 25]. Available from: https://www.prepwatch.org/country/uganda/

[11] Galea JT, Kinsler JJ, Salazar X, Lee S-J, Giron M, Sayles JN (2011). Acceptability of pre-exposure prophylaxis as an HIV prevention strategy: barriers and facilitators to pre-exposure prophylaxis uptake among at-risk Peruvian populations. Int J STD AIDS.

[12] Jayakumaran JS, Aaron E, Gracely EJ, Schriver E, Szep Z (2016). Knowledge, attitudes, and acceptability of pre-exposure prophylaxis among individuals living with HIV in an urban HIV clinic. Clark JL, editor. PLoS One.

[13] MTN VOICE 003 (2015). Final results of the HIV prevention study VOICE are published in NEJM | Microbicide Trials Network.

[14] Escudero DJ, Kerr T, Wood E, Nguyen P, Lurie MN, Sued O (2015). Acceptability of HIV pre-exposure prophylaxis (PREP) among people who inject drugs (PWID) in a CANADIAN setting. AIDS Behav.

[15] Yi S, Tuot S, Mwai GW, Ngin C, Chhim K, Pal K (2017). Awareness and willingness to use HIV pre-exposure prophylaxis among men who have sex with men in low- and middle-income countries: a systematic review and meta-analysis. J Int AIDS Soc.

[16] Avert. New HIV infections fall by more than half in Ugandan fishing communities: Avert; 2019. [cited 2021 Sep 7]. Available from: https://www.avert.org/news/new-hiv-infections-fall-more-half-ugandan-fishing-communities

[17] Kwagonza L, Bulage L, Okello PE, Kusiima J, Kadobera D, Ario AR (2020). Comprehensive knowledge of HIV prevention among fishing communities of Lake Kyoga, Uganda, 2013. BMC Public Health.

[18] UBOS (2016). Kampala Profiles: Makindye.

[19] USAID (2010). HIV prevalence estimates from the demographic and health surveys: updated June 2010. ICF Macro.

[20] Kiwanuka N, Ssetaala A, Nalutaaya A, Mpendo J, Wambuzi M, Nanvubya A (2014). High incidence of HIV-1 infection in a general population of fishing communities around Lake Victoria, Uganda. PLoS One.

[21] UPHIA (2019). Uganda Population-Based Hiv Impact Assessment (Uphia) 2016-2017.

[22] Fleiss JL, Levin B, Paik MC (2003). Statistical methods for rates and proportions.

[23] Frankis JS, Young I, Lorimer K, Davis M, Flowers P (2016). Towards preparedness for PrEP: PrEP awareness and acceptability among MSM at high risk of HIV transmission who use sociosexual media in four Celtic nations: Scotland, Wales, Northern Ireland and the Republic of Ireland: an online survey. Sex Transm Infect.

[24] Eisingerich AB, Wheelock A, Gomez GB, Garnett GP, Dybul MR, Piot PK (2012). Attitudes and acceptance of Oral and parenteral HIV Preexposure prophylaxis among potential user groups: a multinational study. PLoS One.

[25] Kuteesa MO, Quaife M, Biraro S, Katumba KR, Seeley J, Kamali A (2019). Acceptability and predictors of uptake of anti-retroviral pre-exposure prophylaxis (PrEP) among fishing communities in Uganda: a cross-sectional discrete choice experiment survey. AIDS Behav.

[26] Koss CA, Charlebois ED, Ayieko J, Kwarisiima D, Kabami J, Balzer LB (2020). Uptake, engagement, and adherence to pre-exposure prophylaxis offered after population HIV testing in rural Kenya and Uganda: 72-week interim analysis of observational data from the SEARCH study. Lancet HIV.

[27] Torres TS, Konda KA, Vega-Ramirez EH, Elorreaga OA, Diaz-Sosa D, Hoagland B (2019). Factors associated with willingness to use pre-exposure prophylaxis in Brazil, Mexico, and Peru: web-based survey among men who have sex with men. JMIR Public Health Surveill.

[28] Mayer KH, Agwu A, Malebranche D (2020). Barriers to the wider use of pre-exposure prophylaxis in the United States: a narrative review. Adv Ther.

[29] Mack N, Odhiambo J, Wong CM, Agot K (2014). Barriers and facilitators to pre-exposure prophylaxis (PrEP) eligibility screening and ongoing HIV testing among target populations in Bondo and Rarieda, Kenya: results of a consultation with community stakeholders. BMC Health Serv Res.

[30] Nideröst S, Gredig D, Hassler B, Uggowitzer F, Weber P (2018). The intention to use HIV-pre-exposure prophylaxis (PrEP) among men who have sex with men in Switzerland: testing an extended explanatory model drawing on the unified theory of acceptance and use of technology (UTAUT). J Public Health.

[31] Muhumuza R, Ssemata AS, Kakande A, Ahmed N, Atujuna M, Nomvuyo M (2021). Exploring perceived barriers and facilitators of PrEP uptake among Young people in Uganda, Zimbabwe, and South Africa. Arch Sex Behav.

